# Loperamide Induced Life Threatening Ventricular Arrhythmia

**DOI:** 10.1155/2016/5040176

**Published:** 2016-07-28

**Authors:** Ankit Upadhyay, Vijaykumar Bodar, Mohammad Malekzadegan, Sharanjit Singh, William Frumkin, Aditya Mangla, Kaushik Doshi

**Affiliations:** Jamaica Hospital Medical Center, Department of Medicine, Jamaica, NY 11418, USA

## Abstract

Loperamide is over-the-counter antidiarrheal agent acting on peripherally located *μ* opioid receptors. It is gaining popularity among drug abusers as opioid substitute. We report a case of a 46-year-old male that was presented after cardiac arrest. After ruling out ischemia, cardiomyopathy, pulmonary embolism, central nervous system pathology, sepsis, and other drug toxicity, we found out that patient was using around 100 mg of Loperamide to control his chronic diarrhea presumably because of irritable bowel syndrome for last five years and consumed up to 200 mg of Loperamide daily for last two days before the cardiac arrest. We hypothesize that the patient's QTc prolongation and subsequent cardiac arrest are due to Loperamide toxicity. Patient experienced gradual resolution of tachyarrhythmia and gradual decrease in QTc interval during hospitalization which supports the evidence of causal relationship between Loperamide overdose and potentially fatal arrhythmias. It also provided the clue that patient may have congenital long QT syndrome which was unmasked by Loperamide causing ventricular arrhythmias. This case adds one more pearl in the literature to support that Loperamide overdose related cardiac toxicity does exist and it raises concerns over Loperamide abuse in the community.

## 1. Introduction

Drug overdose related cardiac toxicity and arrhythmia are well described in the literature and various drugs are responsible for causing life threatening ventricular arrhythmias [1]. Loperamide is over-the-counter antidiarrheal agent acting on peripherally located *μ* opioid receptors. We report an uncommon potentially fatal side effect of Loperamide ingestion in massive dose, a case of life threatening ventricular arrhythmia associated with Loperamide overdose.

## 2. Case Presentation

**Figure 1:**
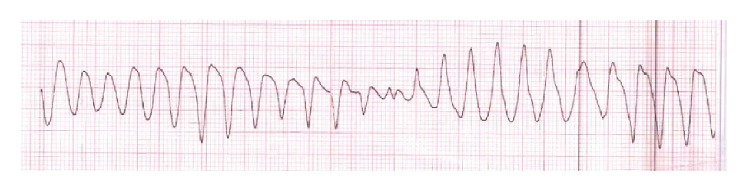
Torsades de pointes ventricular tachycardia.

**Figure 2:**
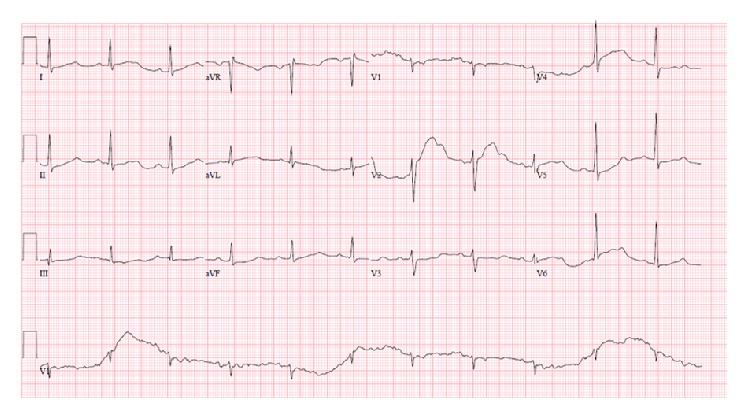
Sinus rhythm, nonspecific ST-T wave abnormality, and prolonged QTc of 620 msec.

**Figure 3:**
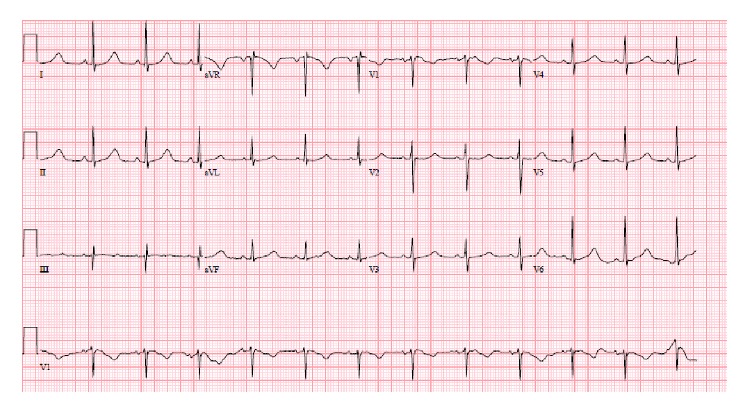
Sinus rhythm and prolonged QTc of 472 msec, several days after stopping Loperamide.

**Figure 4:**
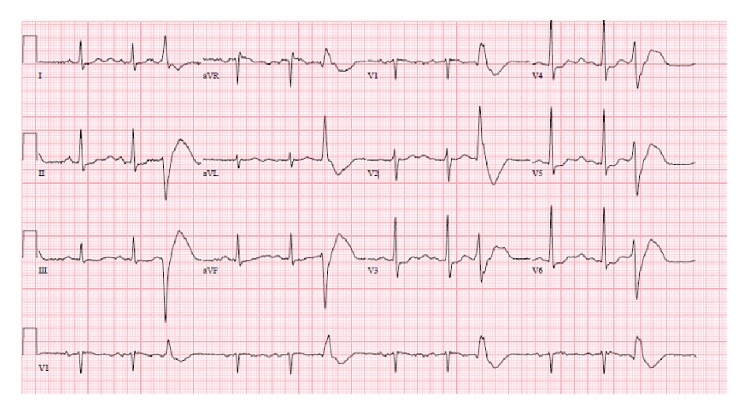
Sinus rhythm, premature ventricular complexes, and prolonged QTc of 456 msec, several days after stopping Loperamide.

**Figure 5:**
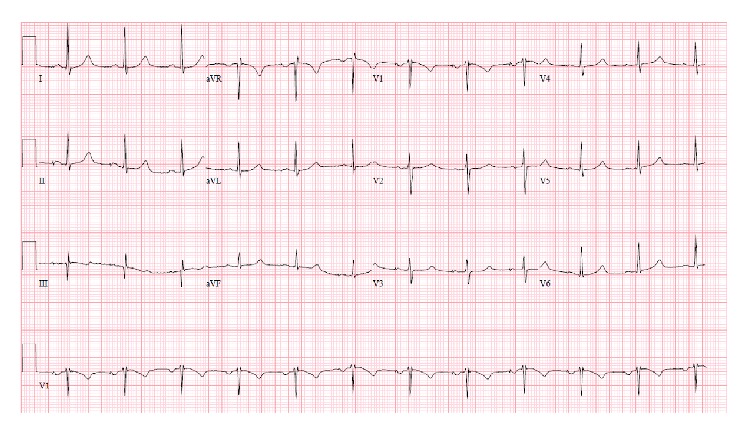
Sinus rhythm and QTc of 432 msec, off Loperamide.

46-year-old male was presented in the ER after cardiac arrest. Patient had two-hour flight before he started feeling light-headed and passed out at the airport. The airport emergency services found him in ventricular fibrillation and provided him with two shocks before he regained consciousness. Subsequent EKG showed normal sinus rhythm and prolonged QT (QTc-589 msec); EKG is not available. Upon arrival to ER, patient was alert and oriented but amnesic about past events. Patient was taken for cardiac catheterization which revealed nonobstructive coronaries and normal ejection fraction. Electrolytes were unremarkable except potassium of 3.1 which was replaced. Complete blood counts, renal function, serum troponin, and serum magnesium were within normal limits. MRI of brain revealed no significant abnormalities. Shortly after coronary angiogram, patient again went to cardiac arrest and resuscitated with one shock. Cardiac monitor showed torsades de pointes ventricular tachycardia (Figure 1) with hemodynamic instability. Patient was intubated for airway protection and received IV magnesium and IV amiodarone. Toxicology screen was unremarkable except benzodiazepine he received during intubation (midazolam) for sedation. EKG showed normal sinus rhythm, nonspecific ST-T wave abnormality, and prolonged QT (QTc-620 msec) (Figure 2). Echocardiogram revealed structurally normal heart. Acute pulmonary embolism was also ruled out with computed tomography pulmonary angiogram. Patient's spouse reported that patient has been taking large amount of Loperamide for irritable bowel syndrome for more than 5 years. However, she was not able to quantify the amount of Loperamide. Patient persistently experienced multiple episodes of sustained ventricular tachycardia requiring multiple defibrillation in setting of significantly prolonged QTc (>600 msec) on multiple serial EKGs. Patient was treated with metoprolol, lidocaine, and magnesium infusion. Over the period of 7 days, patient stopped having sustained ventricular tachycardia. Frequency of nonsustained ventricular tachycardia was also reduced and patient was extubated after several days of CCU monitoring. Lidocaine and magnesium were discontinued and metoprolol was continued. Loperamide levels were not obtained. After extubation, patient denied having similar episode in the past, chest pain, palpitations, syncope, shortness of breath, and any family history of sudden cardiac death or cardiac diseases. However, patient admitted that he has been self-medicating with over-the-counter 2 mg Loperamide approximately 50 tablets a day for diarrhea predominant irritable bowel syndrome for more than 5 years. He also admitted having approximately 100 pills of Loperamide daily for the last 2 days before admission. Since patient was buying over-the-counter Loperamide from different pharmacy; we could not determine exact frequency of purchase. Patient denied using illicit drug use, herbal supplements, or concurrent p-glycoprotein inhibitors. Serial EKG monitoring showed gradual decrease in QTc. However, serial EKG monitoring off Loperamide for several days revealed normal sinus rhythm and QTc of 472 msec (Figure 3) and 456 msec (Figure 4). Only one EKG revealed QTc of 432 msec (Figure 5). Thus, patient's QTc interval did not completely normalize after several days off all QTc prolonging medications and subsequently he received dual chamber automated cardioverter defibrillator (AICD) before discharge for secondary prevention of cardiac arrhythmia related death. Since majority of patient's QTc interval was >440 msec even after a week of stopping all QT prolonging medications, at this time, we think that patient may have subclinical long QT syndrome which was unmasked by Loperamide. However, electrophysiological studies were not performed to verify the diagnosis. Patient was instructed not to use Loperamide again.

## 3. Discussion

Loperamide has been increasingly recognized as opiate substitute. In a web based study, Daniulaityte et al. concluded that large doses of Loperamide are being used among drug abusers to alleviate opioid withdrawal symptoms [2]. There have been few case reports of life threatening ventricular tachycardia including torsades de pointes attributed to Loperamide overdose [3–7]. Dosage of Loperamide varied in all cases reported ranging from 60 mg up to 400 mg daily, which were dangerously exceeding recommended dose of 16 mg per day. All reported cases had significantly prolonged QTc. QTc prolongation occurs due to disturbances of repolarization activity of cardiac muscle. Although numbers of hypotheses exist in the current literature, the exact mechanism of Loperamide causing QTc prolongation is unknown till date. At therapeutic doses, Loperamide acts on peripheral *μ* receptors to produce antidiarrheal effects. Moreover, Reynolds et al. [8] described that Loperamide induces verapamil-like effect by blocking calcium channels in animal models. At extremely high doses, Loperamide may produce cardiac toxicity by delaying repolarization [5]. In one in vitro study, methadone and terfenadine are linked to QTc prolongation via inhibition of cardiac human ether-a-go-go potassium channel [9, 10]. Loperamide demonstrates structural similarities with methadone and terfenadine by having multiple phenyl rings [9]. Therefore, it can be assumed that Loperamide exerts its QTc prolongation effect and ultimately cardiac arrhythmias through similar mechanism. However, integrity of this proposed mechanism is yet to be verified. Since there is paucity of the data in the literature regarding Loperamide related cardiac toxicity, it is unclear at what doses Loperamide starts affecting the cardiac action potential.

Based on patient's history of chronic Loperamide use in excessive amount (no evidence of other causes of patient's presentation), we hypothesize that there is a temporal relationship between Loperamide abuse and ventricular arrhythmias. During serial monitoring of QTc interval, a gradual decrease in QTc interval to borderline level also indicates that patient may have subclinical congenital long QT syndrome which was unmasked by the QTc prolonging drug like Loperamide resulting in life threatening ventricular arrhythmias such as torsades de pointes in our patient.

## 4. Conclusion

Although there is no robust evidence regarding Loperamide induced cardiovascular toxicity, few case reports established temporal association between Loperamide overdose and ventricular tachyarrhythmias. If Loperamide is used in massive doses, it can induce arrhythmias that can be fatal. General population and clinicians should be aware of this potentially dangerous medication and its abuse potential. Future studies should focus on mechanism by which Loperamide causes the cardiac toxicity and dose response relationship.
